# Severity and location of lumbar spine stenosis affects the outcome of total knee arthroplasty

**DOI:** 10.1186/s13018-021-02864-x

**Published:** 2021-12-20

**Authors:** William L. Sheppard, Kevin M. McKay, Alexander Upfill-Brown, Gideon Blumstein, Howard Y. Park, Akash Shah, Adam A. Sassoon, Don Y. Park

**Affiliations:** 1 Department of Orthopedic Surgery, University of California, Los Angeles, 1250 16th St Suite 2100, Santa Monica, CA 90404 USA; 2 David Geffen School of Medicine, University of California, Los Angeles, 10833 Le Conte Ave, Los Angeles, CA 90095 USA

**Keywords:** Spinal stenosis, Total knee arthroplasty, Outcomes

## Abstract

**Background:**

Recent studies have noted that patients with pre-existing lumbar spinal stenosis (LSS) have lower functional outcomes after total knee arthroplasty (TKA). Given that LSS manifests heterogeneously in location and severity, its influence on knee replacement merits a radiographically targeted analysis. We hypothesize that patients with more severe LSS will have diminished knee mobility before and after TKA.

**Methods:**

This retrospective case series assessed all TKAs performed at our institution for primary osteoarthritis from 2017–2020. Preoperative lumbar magnetic resonance image (MRI) with no prior lumbar spine surgery was necessary for inclusion. Stenosis severity was demonstrated by (1) anterior–posterior (AP) diameter of the thecal sac and (2) morphological grade. TKA outcomes in 103 cases (94 patients) were assessed by measuring preoperative and postoperative arc of motion (AOM), postoperative flexion contracture, and need for manipulation under anesthesia.

**Results:**

Patients with mild stenosis did significantly better in terms of postoperative knee AOM. As AP diameter decreased at levels L1–2, L2–3, L3–4, and L4–5, there was a significant reduction in preoperative-AOM (*p* < 0.001 for each), with a 16 degree decrease when using patients’ most stenotic level (*p* < 0.001). The same was noted with respect to increased morphological grade (*p* < 0.001), with a 5 degree decrease for patients’ most stenotic level (*p* < 0.001).

**Conclusion:**

Severe LSS, which is readily demonstrated by a reduction in the AP diameter of the thecal sac or increased morphological grade on MRI, correlated with a significant reduction in preoperative AOM that was not improved after TKA. Persistent postoperative reductions in AOM may contribute to reduced patient satisfaction and recovery.

*Level of evidence*: Level 4

## Background

The relationships between the knee, hip, and spine have garnered interest, as pathology in one location often manifests over time as pain, deformity, or degeneration in another [1–5]. The rising incidence of knee and hip arthroplasty in an aging population [6] has provided an opportunity to further characterize these relationships and their effect on outcomes after surgical correction [7–10]. The prevalence of lumbar spinal stenosis (LSS) is higher in these populations as well; thus, there is an increased interest in how LSS affects total hip and knee arthroplasty outcomes when performed for primary osteoarthritis (OA) [8, 10–13].

Recent studies suggest that in patients undergoing total hip arthroplasty (THA), those with concomitant LSS appear to have worse functional outcomes, patient satisfaction scores, and activity levels when compared to patients without stenosis [11]. Additionally, there is evidence that in patients who undergo both THA and decompression for LSS, those who undergo lumbar decompression prior to THA have higher health-related quality of life (HRQOL) scores postoperatively [12]. Total knee arthroplasty (TKA) is also affected by prior diagnosis of LSS. Patients with stenosis who underwent TKA were found to have lower Knee Society postoperative functional outcome scores [13]. Prior TKA was also identified as an independent risk factor for poor outcomes 1 year after surgical decompression for LSS [9].

While the aforementioned studies elucidate a strong relationship between LSS and outcomes in lower extremity arthroplasty, they fail to explore whether the heterogeneity in severity and location of LSS differentially contributes to these poor reported results. Specifically, LSS can vary in morphology (central or lateral), present at any level of the lumbar spine, and differ in radiographic severity [14, 15]. Given this clinical variability, a more targeted examination may improve prognostic accuracy, allowing surgeons to better inform their patients’ expectations preoperatively and to understand the potential consequences postoperatively [16, 17].

The purpose of this study was to expand upon potential relationships between lumbar stenosis and TKA, focusing on clinically relevant outcome measures such as Arc of Motion (AOM), Range of Motion (ROM), and need for Manipulations Under Anesthesia (MUA). We additionally sought to elucidate a potential relationship between the level of stenosis and the outcome measures listed above. We hypothesized that in patients undergoing TKA, decreases in both pre- and postoperative knee mobility would directly correlate with increasing severity and location of LSS.

## Methods

### Study design

After Institutional Review Board approval, all TKAs at a single healthcare system between 2017 and 2020 were identified. A total of 933 consecutive TKAs in 845 patients were performed for OA [3]. Of this cohort, we identified 103 TKAs performed in 94 patients who met the following inclusion criteria: pre-operative lumbar MRI, no prior history of lumbar spine surgery, pre- and post-operative knee mobility measurements, and at least 3 months of postoperative follow-up. Patients were excluded for not possessing preoperative lumbar spine MRI (706), prior lumbar spine surgery (57), inadequate follow-up with pre- and post-operative knee mobility measurements (30), and for the presence of severe scoliosis or suspected secondary OA based on chart review (37).

### Quantitative/qualitative grading of MRIs

**Fig. 1:**
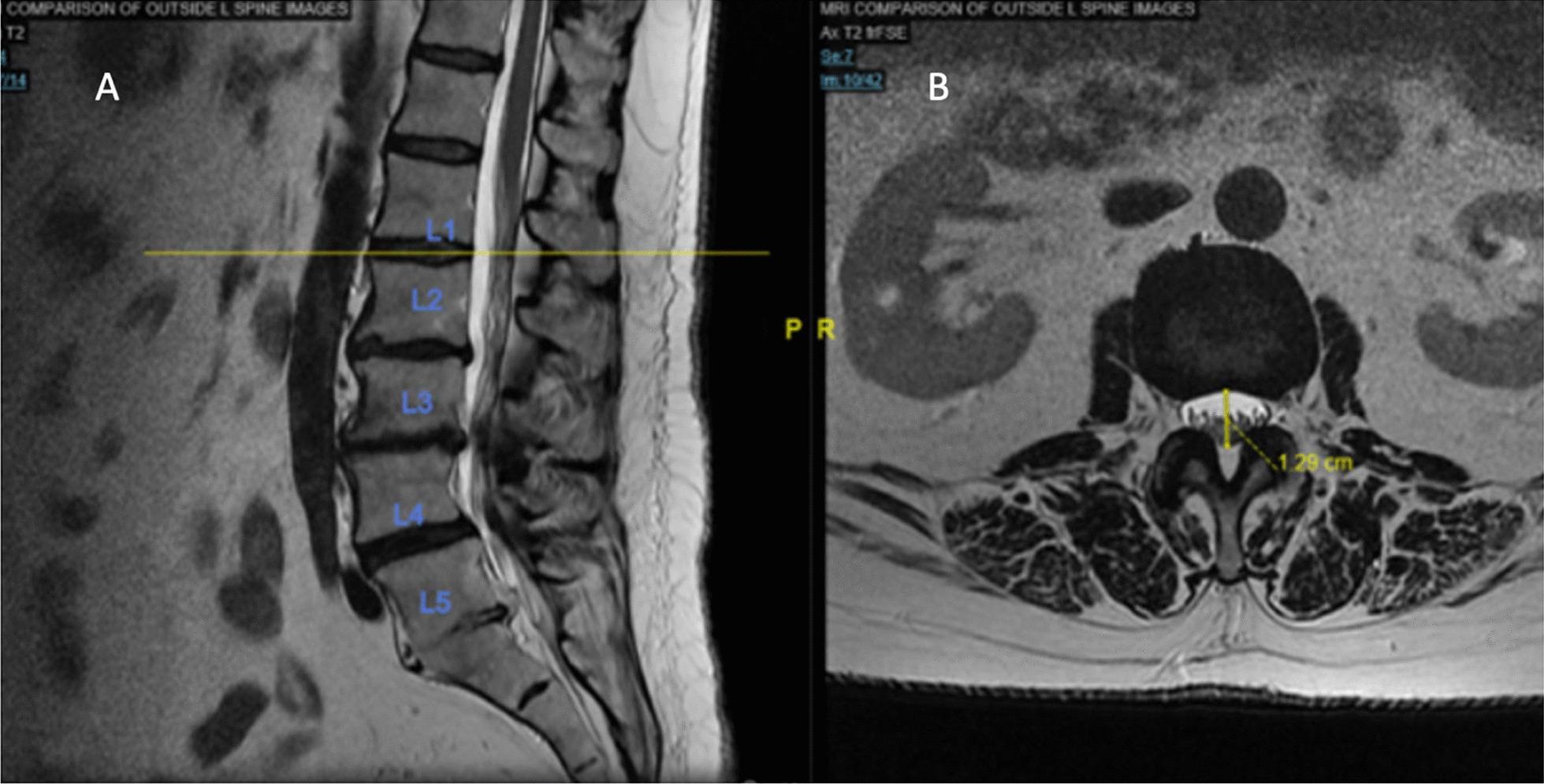
Anterior–posterior (AP) diameter. **A** Sagittal T2-weighted MRI image at the intervertebral disc space between lumber level 1 and 2. **B** The corresponding T2-weighted axial MRI image from which the AP diameter measurement is shown. A line drawn from anterior-most aspect of the thecal sac to the most posterior aspect yields the measurement needed to assess stenosis in the AP plane

**Fig. 2:**
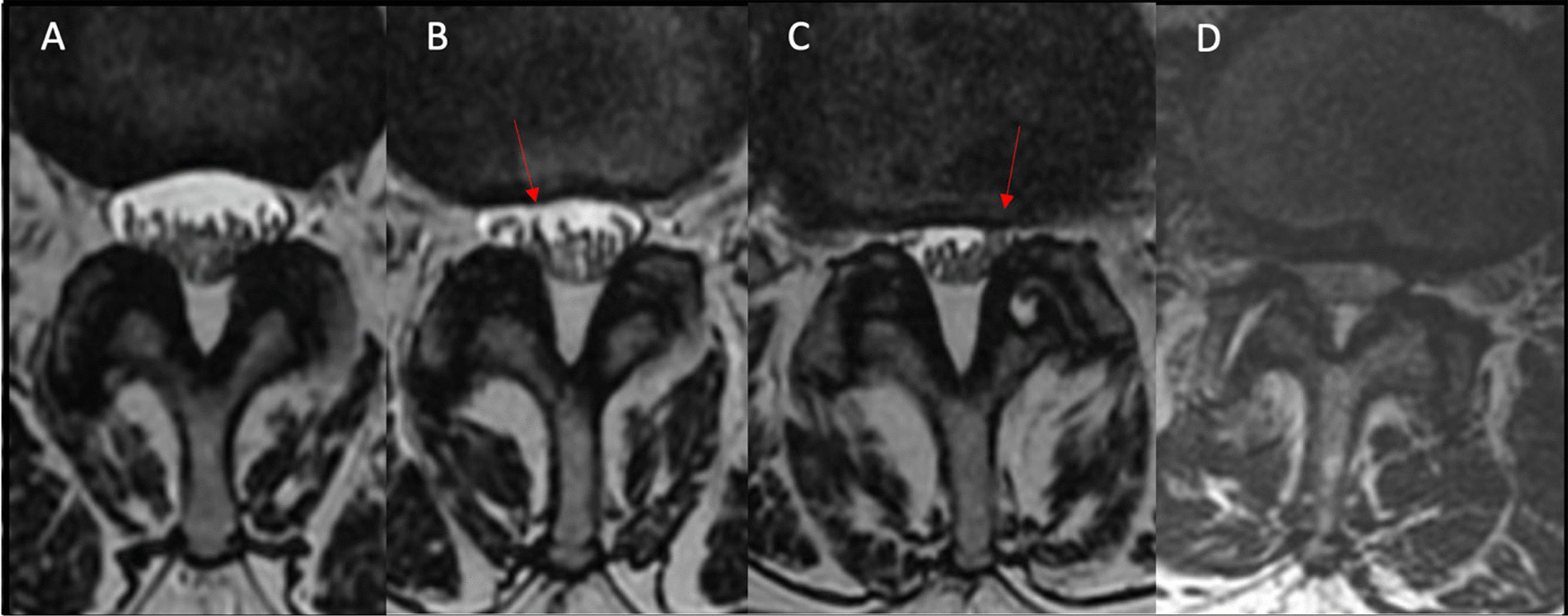
Morphological grade of stenosis [17, 20]. Axial T2-weighted MRI images shown. **A** Grade 0, normal thecal sac without cerebral spinal fluid (CSF) effacement. **B** Grade 1, mild CSF effacement without significant nerve root crowding/compression. **C** Grade 2, moderate CSF effacement with nerve root crowding/compression. **D** Grade 3, severe CSF effacement with indistinguishable individual nerve roots due to significant crowding/compression

Measurements of LSS severity were performed on T2-weighted lumbar MRIs by 2 researchers independently. Severity was evaluated by (1) decreased AP diameter of the thecal sac [16, 18–20], demonstrated in Fig. 1A, B, and (2) increased morphological grade from L1–S1 as described by Guen et al. and demonstrated in Fig. 2A–D [17, 21]. Interrater reliability for (1) was assessed by calculating a correlation coefficient (0.96) and for (2) using Cohen’s kappa coefficient (kappa = 0.82, *p* < 0.05) [22].

### Demographics

Patient demographics including age, sex, and body mass index (BMI) were noted. Potential preoperative confounders were also obtained for regression analyses, which included knee mobility, opiate use, and concurrent comorbidities (diabetes, hypertension, osteoporosis, osteopenia, and nicotine use). Presence or absence of symptomatic LSS was identified through retrospective chart review for documented history of unilateral or bilateral “sciatica”, “back pain”, and/or lower extremity “radiculopathy”. Asymptomatic patients with a confirmed diagnosis of lumbar stenosis were also included in the analysis given the lack of defined correlation between imaging and symptom severity [23].

### TKA outcomes

Primary TKA outcomes measured by attending surgeons, orthopedic residents, and physician assistants during pre- and post-operative office visits were gathered during retrospective chart review, including need for subsequent MUA, pre- and post-operative AOM (defined as the magnitude in degrees of knee flexion minus knee extension), presence of a flexion contracture, and the difference between pre-operative and post-operative AOM (∆AOM). Measurements of continuous variables listed above represent active motion in that the subjects achieved these ranges without the assistance of the medical practitioners above. Discontinuous variables such as presence of flexion contracture or need for MUA were documented by medical practitioners accordingly.

### Statistical analysis

Incidence of the aforementioned comorbidities was reported as averages with 95% confidence intervals (CI). Multivariate regression was used to explore the functional relationship between AP diameter, morphological grade, and the outcomes of interest while controlling for confounding variables such as age, sex and BMI. Significance was determined as *p* < 0.05. Analysis was conducted using R version 3.3.1. (R Core Team. R Foundation for Statistical Computing. Vienna, Austria).

## Results

### Demographics

A total of 94 patients (103 operated upon knees) who underwent TKA for primary OA were included. Of these, the majority were female (73 females to 21 males), with an average BMI of 30.7, and mean age of 71.3 years. Regarding comorbidities, 22% of patients had diabetes mellitus, 73% had hypertension, 79% had osteopenia or osteoporosis, 4% were tobacco smokers, and 38% used opioids pre-operatively (Table 1). Average follow-up was 32 weeks (95% CI 27–36 weeks). In total, 23 patients had history of bilateral sciatic and lower back pain, 54 had unilateral sciatic and lower back pain, 21 had isolated lower back pain, and 5 were asymptomatic.

**Table 1:** Demographics and comorbidities

Demographics and comorbidities	
Average age (years)	71.3
Male (%)	24.5
Average BMI (kg/m^2^)	30.7
Diabetes mellitus (%)	22.3
Hypertension (%)	72.3
Osteoporosis/osteopenia (%)	21.2
Current smoker (%)	4.3
Preoperative opioid use (%)	38.3

### MRI measurements

The average thecal sac AP diameter measured at intervertebral lumbar levels L1–2, L2–3, L3–4, L4–5, and L5–S1 is depicted in Table 2. The most stenotic levels in the AP plane were L3–4 and L4–5. Morphological grade was assessed at the same lumbar levels above. Table 3 depicts the number of subjects and the corresponding morphological grade at each level. The most stenotic levels were L2–3 and L3–4 on average. These levels accounted for 56.2% of morphological stenosis Grade 1 or more. The most stenotic level for both AP diameter and morphological grade overall was L3–4.

**Table 2:** Stenosis measured by AP diameter

Level	Mean (cm)	SD (cm)	95% CI (cm)
L1–2	1.43	0.25	1.39–1.48
L2–3	1.29	0.31	1.23–1.35
L3–4	1.12	0.32	1.04–1.17
L4–5	1.12	0.36	1.05–1.19
L5–S1	1.38	0.37	1.31–1.45

**Table 3:** Morphological grade of stenosis

Level	Grade 0	Grade 1	Grade 2	Grade 3
L1–2	77	20	6	0
L2–3	64	26	12	1
L3–4	51	20	28	4
L4–5	59	21	19	4
L5–S1	102	1	0	0

### TKA outcomes

The mean preoperative AOM was 111.9 degrees (95% CI 109.5–114.4), while the mean postoperative AOM was 117.5 (95% CI 115.3–119.7). The mean ∆AOM was ~ 10.7 degrees (95% CI 8.41–13.0). A total of 9 (8.74%) subjects required MUA, and 20 (19.42%) knees had flexion contracture of at least 5 degrees from terminal extension. Patients with flexion contracture averaged 109.6 degrees of postoperative AOM (95% CI 104.78–114.32). Patients underwent MUA within 90 days either for difficulty with achieving preoperative flexion (7 of 9 patients, 77.78%), or difficult with both flexion and extension (2 of 9 patients, 22.23%).

### Regression analysis

Using the worst level of stenosis (classified by AP Diameter), linear models accounting for preoperative use of opioids, BMI, age, sex, presence of osteopenia, hypertension, or diabetes mellitus (DM) showed a slightly higher incidence of MUA in patients with worse AP diameter (OR 7.11, 95% CI 1.03, 49.15, *p* = 0.045), and a higher postoperative AOM if patients’ worst level of stenosis was less severe (regression coefficient: 9.13, *p* = 0.037; Table 4). Furthermore, a larger preoperative AOM was correlated with less AP stenosis at all levels except L5–S1. Similarly, with respect to preoperative AOM, there was strong correlation with less morphological grade stenosis at every intervertebral disc space except L5–S1 (Table 5). Specifically, there was a significant reduction in preoperative AOM (*p* < 0.001 for each), with a 16-degree decrease when using patients’ most stenotic level measured by AP diameter (*p* < 0.001). The same was noted with respect to increased morphological grade (*p* < 0.001), with a 5-degree decrease for the patients’ most stenotic level (*p* < 0.001).

**Table 4:** Postoperative arc of motion vs AP diameter

Intervertebral level	Regression coefficient (p value)
L1–2	5.21 (0.32)
L2–3	3.22 (0.45)
L3–4	7.67 (0.055)
L4–5	3.75 (0.27)
L5–S1	2.23 (0.47)
Worst level of stenosis	9.13 (0.037)**

**Statistically significant

**Table 5:** Preoperative arc of motion vs AP diameter/morphological grade

Intervertebral level	AP diameter regression coefficient (p value)	Morphological grade regression coefficient (p value)
L1–2	17.64 (< 0.001)**	7.18 (< 0.001)**
L2–3	13.57 (< 0.001)**	6.76 (< 0.001)**
L3–4	12.37 (0.001)**	5.06 (< 0.001)**
L4–5	10.93 (< 0.001)**	4.24 (< 0.001)**
L5–S1	4.47 (0.15)	4.67 (0.70)
Worst level of stenosis	15.76 (< 0.001)**	5.20 (< 0.001)**

**Statistically significant

There were no significant relationships between the incidence of MUA or flexion contracture and grade of stenosis in linear models accounting for preoperative use of opioids, BMI, age, sex, presence of osteopenia, hypertension, and DM. There were also no significant relationships between postoperative AOM and morphological grade at any level. Using the worst level of stenosis (classified by morphological grade), when assessing ∆AOM, patients with higher morphological grades of stenosis counterintuitively had better postoperative ∆AOM at L2–3 (*p* = 0.02), and a trend in the same direction at their worst level of stenosis (*p* = 0.06).

## Discussion

This case series demonstrates a correlation between severity of lumbar spine stenosis and knee mobility/function in a group of patients who subsequently underwent total knee arthroplasty for primary osteoarthritis. In elucidating this relationship, it gives credence to the variability of spinal stenosis by utilizing radiographic measures to stratify LSS in severity and location (specific lumbar intervertebral disc space), providing a more targeted analysis.

For both measures of stenosis severity, namely (1) increasing morphological grade and (2) decreasing AP diameter, patients demonstrated reduced preoperative AOM when stenosis was noted at any level of the lumbar spine aside from L5–S1. This relationship was inversely proportional. As the morphological grade of stenosis increased (worsened) or AP diameter of the thecal sac lessened, a correlation was noted with reductions in preoperative AOM at all levels except L5–S1. This previously undocumented relationship between stenosis severity and poor preoperative knee mobility was most significant when assessing each patient’s worst/most stenotic level (Table 5), further supporting our hypothesis that this deficit was related to LSS. At the worst level of stenosis (most often L3–4), 16- and 5-degree reductions in preoperative AOM were noted when assessed by AP diameter and morphological grade, respectively (*p* < 0.001). At L5–S1, our series revealed a lack of stenosis by both utilized measures, which can be explained by increased anatomical size of the spinal canal at this level on average, and less nerve rootlets within the thecal sac [17, 20].

Reduced preoperative knee mobility has previously been shown to be associated with decreased functional outcomes following TKA [24]. However, there are limited data on the effect of optimizing preoperative knee mobility in the sagittal plane prior to TKA [25]. This study supports the importance of evaluating patients for the presence of factors (including LSS) that may contribute to decreased preoperative AOM. In this series, patients did not improve significantly from the preoperative AOM assessment, regaining approximately 10 degrees on average postoperatively. This result emphasizes that determining the etiology of poor preoperative AOM is paramount, as these deficits may not be correctable by TKA alone.

Additionally, postoperative AOM was significantly reduced at the level of most significant stenosis in the AP plane (Table 4), which was located at either L2–3 or L3–4 in nearly 60% of subjects overall. These levels contribute most significantly to quadricep functionality when the L3 nerve root is affected, which could explain the increased incidence of postoperative MUA when these levels are affected. Furthermore, in a seemingly counterintuitive manner, patients with worse grade of stenosis at L2–3 had better recovery of knee mobility following TKA. Stenosis at this level may have had less effect on the L3 nerve root, allowing for better postoperative rehabilitation of the quadriceps.

While prior work in this area is limited, there are important comparisons to be made with our study that may guide future investigations. Additionally, several studies have noted similar correlations between TKA and lumbar stenosis to a lesser extent. Pivec et al. investigated outcomes of TKA (Knee Society objective scores, function scores, range of motion, radiographic outcomes, and implant survivorship) in 115 patients with LSS, finding significantly lower mean postoperative Knee Society function and objective scores compared to control patients without stenosis [13]. However, they neither found postoperative differences in knee range of motion between study groups, nor preoperative difference between LSS and non-LSS cohorts. This incongruity may be attributed to differences in selection criteria used for their study, which required patients in the LSS cohort to have an official diagnosis of LSS or prior surgery for LSS, possibly enhancing preoperative knee range of motion [13]. Our study excluded patients with prior surgery for LSS for several reasons. Corrective surgery for lumbar degenerative disease (LDD) has been shown to increase failure rates in TKA above that observed in patients with LDD alone [8]. Further, our study relies on preoperative MRI analysis as we attempted to negate postoperative MRI degradation as well as occult postoperative epidural scarring that could confound our findings [26]. This approach allows for a more nuanced investigation of the isolated effect of LSS on knee mobility surrounding TKA.

There are several limitations to this study which include the following. (1) The retrospective design of this study, which relies on chart review for collection of data on patient symptoms. (2) The methods by which we define stenosis are based on several articles [16–20]. While our observers achieved acceptable interrater reliability coefficients, providing confidence in the MRI measurements, these methods lack normalization to take into account variations in normal subject anatomy. While there are several studies offering additional methodologies for radiographic grading of LSS [15, 17, 27–29] and attempting to define population parameters [21], there is a lack of consensus regarding MRI measurements and their true significance as prognostic indicators [16, 23, 30–32]. Regarding the particular methodology we utilized in grading LSS, the authors found some deficiencies in its use. Namely, some patients have mild decrease in anterior CSF space (very little to no obliteration), but do have some clumping of the cauda equina, making it difficult to distinguish grades 1 and 2 as currently defined by Guen, Y. L. et al. [17]. (3) There is a lack of patient reported outcome scores to correlate with the functional outcomes stated above. This is another inherent limitation of the retrospective nature of this study as > 80% of subjects lacked patient reported outcome scores. The effect of patient-reported LSS symptom severity scores on TKA outcomes is an important future direction. Lastly, some heterogeneity in MRI quality and formatting of axial slices exists due to studies obtained at outside facilities. However, despite these limitations, we believe this study successfully identifies several novel relationships between lumbar stenosis and its effect on TKA functional outcomes through reductions in pre- and postoperative AOM, most influenced by stenosis at levels between L2-L4.

In conclusion, this study supports the evaluation of patients prior to TKA for any signs/symptoms of LSS. Patients with preoperative LSS, especially at levels affecting the L3 nerve root, may have poor pre- and postoperative knee AOM and are at risk for reduced patient satisfaction, function, and overall recovery after TKA. While preoperative AOM was most significantly reduced by concomitant LSS at various levels in our series, these reductions did not improve significantly following TKA, with 9 subjects requiring MUA and 20 subjects developing flexion contracture postoperatively. These findings suggest that a preoperative lumbar spine evaluation may be useful in properly selecting patients who will be candidates for optimal recovery of knee functionality after TKA, as well as adequately discussing expectations and goals with patients before surgery.

## Data Availability

The datasets generated and/or analyzed during the current study are not publicly available due to the regulations by our Institutional Review Board, but can be made available from the corresponding author by request.

## Abbreviations

LSS: Lumbar spinal stenosis
TKA: Total knee arthroplasty
MRI: Magnetic resonance imaging
AP: Anterior–posterior
AOM: Arc of motion
THA: Total hip arthroplasty
HRQOL: Health-related quality of life
OA: Osteoarthritis
BMI: Body mass index
MUA: Manipulation under anesthesia
∆AOM: Difference between preoperative and postoperative AOM
DM: Diabetes mellitus
CSF: Cerebrospinal fluid
